# Molecular and bioengineering strategies to improve alginate and polydydroxyalkanoate production by *Azotobacter vinelandii*

**DOI:** 10.1186/1475-2859-6-7

**Published:** 2007-02-16

**Authors:** Enrique Galindo, Carlos Peña, Cinthia Núñez, Daniel Segura, Guadalupe Espín

**Affiliations:** 1 Departamento de Ingeniería Celular y Biocatálisis, Instituto de Biotecnología, Universidad Nacional, Autónoma de México, Apdo. Post. 510-3 Cuernavaca, 62250, Morelos, México; 2 Departamento de Microbiología Molecular, Instituto de Biotecnología, Universidad Nacional Autónoma, de México, Apdo. Post. 510-3 Cuernavaca, 62250, Morelos, México

## Abstract

Several aspects of alginate and PHB synthesis in *Azotobacter vinelandii *at a molecular level have been elucidated in articles published during the last ten years. It is now clear that alginate and PHB synthesis are under a very complex genetic control. Genetic modification of *A. vinelandii *has produced a number of very interesting mutants which have particular traits for alginate production. One of these mutants has been shown to produce the alginate with the highest mean molecular mass so far reported. Recent work has also shed light on the factors determining molecular mass distribution; the most important of these being identified as; dissolved oxygen tension and specific growth rate. The use of specific mutants has been very useful for the correct analysis and interpretation of the factors affecting polymerization. Recent scale-up/down work on alginate production has shown that oxygen limitation is crucial for producing alginate of high molecular mass, a condition which is optimized in shake flasks and which can now be reproduced in stirred fermenters. It is clear that the phenotypes of mutants grown on plates are not necessarily reproducible when the strains are tested in lab or bench scale fermenters. In the case of PHB, *A. vinelandii *has shown itself able to produce relatively large amounts of this polymer of high molecular weight on cheap substrates, even allowing for simple extraction processes. The development of fermentation strategies has also shown promising results in terms of improving productivity. The understanding of the regulatory mechanisms involved in the control of PHB synthesis, and of its metabolic relationships, has increased considerably, making way for new potential strategies for the further improvement of PHB production. Overall, the use of a multidisciplinary approach, integrating molecular and bioengineering aspects is a necessity for optimizing alginate and PHB production in *A. vinelandii*.

## 1. Background

Alginates form an important family of biopolymers of both technological and scientific interest. These polymers are linear polysaccharides, which are composed of variable amounts of (1–4)-β-D-mannuronic acid and its epimer, α-L-guluronic acid. Alginates present a wide range of applications, acting for example as stabilizing, thickening, gel or film-forming agents, in various industrial fields. Currently, commercial alginates are extracted from marine brown algae and are used for a variety of applications, mainly in the food and pharmaceutical industries [1]. Increasingly new applications are being discovered for these polymers; an example of this is its use as a source of soluble fiber [2].

Alginates extracted from algae are relatively cheap products, having selling prices in the range US$ 5–20/kg for the majority of the applications [1]; however, alginates of very high purity are used in the pharmaceutical field and these are sold for up to US$ 40,000/kg [1]. The algal alginates have several problems concerning their production which may limit their use in many interesting contexts, especially in the pharmaceutical and chemical industries, where polymers with a very well defined composition, are required. Algal alginates are complex mixtures of polymers, exhibiting a wide range of molecular masses and compositions (G-M) and blocks distribution. These characteristics are practically impossible to control using the current procedure, which relies on harvesting algae from the ocean where there is no control over the environmental conditions, which in turn define the molecular characteristics of the polymer.

Alginates are also produced by bacteria and many of their physicochemical characteristics are similar to those of algae, so that they can be used for the same applications as algal alginates, as well as in other more sophisticated contexts. Alginates produced by microorganisms differ from those of algae because bacterial polymers are acetylated [3]. In addition, bacterial alginates usually have a higher molecular mass than the algal polymers (ranging from 48 to 186 kDa). A molecular mass as high as 4,000 kDa for the polymer synthesized by a mutant strain of *A. vinelandii *has been reported [4]. Both acetylation and molecular mass directly affect directly the viscosity and other rheological properties of alginate solutions and, therefore, this would determine its utility in specific applications of alginate in the food and pharmaceutical fields.

In the bacterial world, alginates are produced by *Pseudomonas *and *Azotobacter *species. A considerable amount of work has been published regarding the production of alginate by the *Pseudomonas *species, an interest driven mainly by the fact that alginate plays an important role in the pathogenicity of *Pseudomonas aeruginosa *in cystic fibrosis. In contrast to *P. aeruginosa*, *A. vinelandii *is a non-pathogenic soil bacterium, which can be used for the development of biotechnological process to produce alginate. This characteristic, as well as the interest in the role that alginate plays in cyst formation has motivated the study of various aspects concerning the production of alginate from *Azotobacter*. Furthermore, the availability of the complete sequence of *A. vinelandii *genome has made this bacterium an ideal model of study, from both technological and scientific points of view.

*A. vinelandii *has another interesting characteristic: under unbalanced growth conditions, this bacterium produces poly-β-hydroxybutyrate (PHB), a polymer of the polyhydroxyalcanoates (PHAs) family of polyesters, which are synthesized by a wide range of bacterial and archaeal species to form carbon and energy reserve materials [5]. PHAs are present in the cytoplasm of bacterial cells as water insoluble granules. Besides playing an important role as a reserve polymer, PHB has been implicated in supporting nitrogen fixation [6]. In *A. vinelandii*, PHB is related to the differentiation process this bacterium undergoes in order to produce cysts resistant to desiccation, as numerous granules are present in mature cysts. However, under laboratory conditions, mutants impaired in PHB synthesis formed mature cysts, resistant to desiccation [7].

PHAs have been drawing attention because they are biodegradable and biocompatible thermoplastics, which can be processed to create a wide variety of consumer products, including plastics, films, and fibers. Imperial Chemical Industries (ICI) started the industrial production of these polyesters in 1982 with the trade name of "Biopol" as a biodegradable substitute for some petroleum-derived plastics [8]. Nowadays, Metabolix and the Kaneka Corporation are producing industrial PHAs [5].

Subjects covered by this review include; research concerning the production of alginate and PHB by *A. vinelandii*, particularly aspects which include the molecular regulation of the production of the two polymers, the construction of recombinant strains for producing more or higher quality alginate and, or PHB, the fermentation conditions which result in attractive bioprocess yields and the potential for scaling-up such processes,

## 2. Alginate and PHB synthesis in *A. vinelandii*

### 2.1 Alginate biosynthesis and its regulation

**Figure 1:**
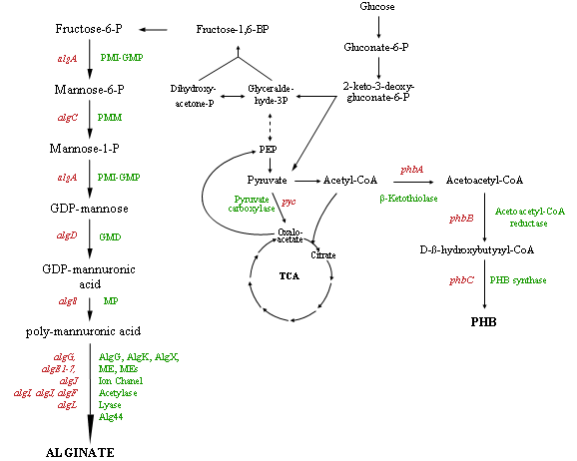
Pathways for alginate and poly-β-hydroxybutyrate (PHB) biosynthesis and their metabolic relations in *Azotobacter vinelandii*. *Dashed arrows *indicate multi-enzyme pathways; the enzyme names and their abreviations are indicated in *green *and the corresponding genes are in *red*. PMI, phosphomannose isomerase; GMP, guanosine diphosphomannose pyrophosphorylase; PMM, phosphomannomutase; GMD, GDP-mannose dehydrogenase; MP, mannuronate polymerase; ME, mannuronate epimerase AlgG; MEs, mannuronate epimerases AlgE1 to 7.

Biosynthetic pathway for alginates (Figure 1) is similar in both the *Azotobacter *and *Pseudomonas *species and has been the subject of a recent review [9]. Briefly, alginate is synthesized from fructose-6-P, which is isomerized by a bifunctional enzyme phosphomannose isomerase/guanosine diphosphomannose pyrophosphorylase, (PMI-GMP or AlgA) in order to produce mannose-6-P; which is in turn converted by phosphomannomutase (PMM or AlgC) into mannose-1-P; PMI-GMP (AlgA) catalyzes the conversion of mannose-1-P to become GDP-mannose; GDP mannose is oxidized by GDP-mannose dehydrogenase (GMD or AlgD) to GDP-mannuronic acid. Polymerization of GDP-mannuronic acid is carried out by the Alg8 protein, a mannuronate polymerase (MP) [10]. The resulting polymannuronic molecule is then modified by an acetylase complex comprising AlgI, AlgV, AlgF proteins (AlgI, AlgJ and AlgF for *P. aeruginosa*), and some of the non-acetylated mannuronate residues are epimerized to guluronate by a mannuronate epimerase (ME or AlgG) and then exported through the outer membrane via the pore-forming protein AlgE (AlgJ in *A. vinelandii*) (Figure 1).

In the case of *A. vinelandii *but not in that of the *Pseudomonas *species, the exported polymer is converted to the final alginate by a family of seven homologous secreted mannuronan C-5 extracellular epimerases (AlgE1-7) [11](Figure 1). These epimerases are essential for the formation of mature cysts, as a mutation which inactivates the Type I secretion system responsible for the export of the AlgE1-7 epimerases produced cysts lacking the intine and exine coats and were therefore unable to survive desiccation. This suggests that the guluronic acid residues in alginate are important for the formation of the alginate coat surrounding the cysts [12]. It is thought that the AlgG AlgK, AlgX and AlgL proteins form a scaffold which guides the polymer through the periplasm, to then be secreted across the outer membrane [13,14]. AlgL is also an alginate lyase enzyme [15]. The idea that AlgL played a role in *A. vinelandii*, concerning the degradion of the alginate capsule during cyst germination was ruled out, as germination was unaffected in an *algL *mutant [16]. The main role of AlgL in the *Pseudomonas *species is to degrade alginates which fail to be exported out of the cell and thus remain in the periplasm [14,17,18].

Although Alg44 was originally considered to be a component of the polymerase complex, it was recently proposed to be a part of the periplasmic scaffold and/or to play a role in bridging Alg8 in the cytoplasmic membrane with AlgE (AlgJ in *A. vinelandii*) [9]. Alg44 protein has a PilZ domain, a putative cyclic diguanosil monophosphate (c-di-GMP) binding domain [19]. c-di-GMP is a novel regulatory molecule identified as a universal secondary messenger in bacteria [20,21]. Thus, the presence of a PilZ domain, suggests a regulatory role for Alg44.

**Figure 2:**
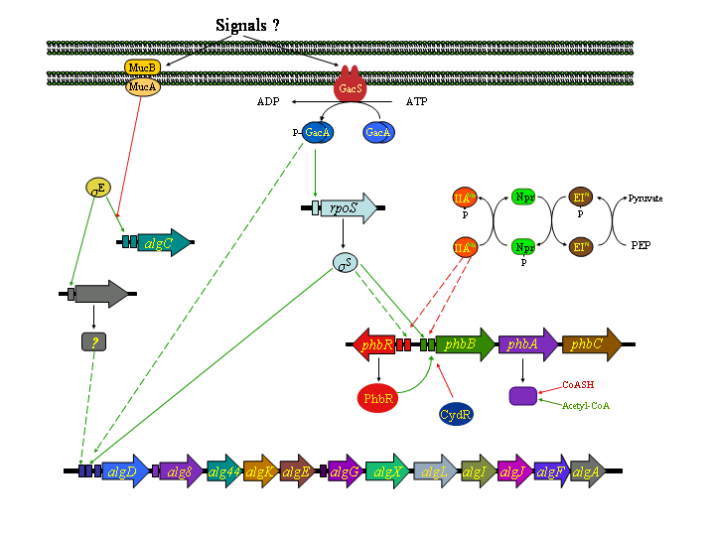
Organization of the *A. vinelandii *alginate and PHB biosynthetic genes. A model for the regulation of the synthesis of these polymers is presented. Green lines: positive regulation; red lines negative regulation; dashed lines indicate unknown intermediates or unknown mechanism of regulation. Promoters are indicated as colored rectangles.

The genes coding for the enzymes of the alginate biosynthesis in *A. vinelandii *have all been identified (Figure 2). With the exception of *algC*, they form the *algD-8-44-K-J-G-X-L-I-V-F-A *cluster [22-28]. Several promoters transcribing this alginate biosynthetic gene cluster have been identified (Figure 2). Three promoters: *algD*p1, *algD*p2 and *algD*p3 located upstream *algD *[22], *alg8*p located upstream *alg8 *[25], and one putative sigma 70 promoter located upstream *algG *[27]. Two promoters were identified upstream *algC*: *algC*p1 and *algC*p2 [28]. Expression of the alginate biosynthetic genes in *A. vinelandii *has been shown to be under the control of the *algUmucABCD *gene cluster, where *algU *codes for the alternative sigma E factor, required for transcription from the *algC*p1 and *alg*Dp2 promoters [28,29]. MucA and MucB proteins act as antisigma E factors (Figure 2). Therefore, mutational inactivation of *algU *results in the impairment of alginate synthesis [30], whereas inactivation of *mucA *leads to alginate overproduction [31]. Expression from the *algD *promoters is also under the control of the two component global regulatory system GacS-GacA, where GacS acts as a sensor histidine kinase protein which phosphorylates GacA, the response regulator that activates transcription of the target genes in its phosphorylated form. Inactivation of *gacS *or *gacA *genes abrogates transcription of *algD *from its three promoters [32,33]. The *rpoS *gene encoding the sigma S factor is under the control of GacA. Inactivation of *rpoS *was shown to impair transcription from the *algD*p1 promoter [33]. Thus, a regulatory cascade which includes the global regulators GacA and RpoS participates in the control of *algD *transcription (Figure 2).

### 2.2. Polyhydroxybutyrate synthesis and its regulation

PHB in *A. vinelandii *is synthesized in three steps from acetyl-CoA [34]. A β-Ketothiolase catalyses the first reaction *i.e*. the condensation of two molecules of acetyl-CoA to form acetoacetyl-CoA, which is reduced by the NADPH dependent acetoacetyl-CoA reductase to produce β-hydroxybutyryl-CoA. PHB synthase catalyses the final reaction: the polymerization of β-hydroxybutyryl-CoA (Figure 1). A PHB biosynthetic gene cluster *phbBAC*, coding for the enzymatic activities β-ketothiolase, acetoacetyl-CoA reductase and PHB synthase respectively has been described in *A. vinelandii *[7,35,36] and *Azotobacter *sp. strain FA8 (Figure 2) [37]. In the same DNA region where *phbBAC *genes are found, other genes related to PHB synthesis were also found: *phbR*, which codes for a member of the AraC family of transcriptional activators; *phbP*, coding for a putative granule-associated protein, and *phbF*, a putative regulator of the *phbP *[7,36]

The regulation of polyhydroxybutyrate synthesis in *A. vinelandii *seems to be complex (Figure 2). In addition to the allosteric control of the first biosynthetic enzyme, β-ketothiolase, by the CoA/acetyl-CoA ratio which was described several years ago [38], other regulatory systems are involved. Transcription of the *phbBAC *biosynthetic operon is initiated from two overlapping promoters, pB1 and pB2. PhbR, encoded by *phbR*, activates transcription of the PHB biosynthetic operon from the pB1 promoter, whereas transcription from pB2 is dependent on the sigma factor RpoS and increases during the stationary phase of growth. Transcription of *phbR *itself also starts from two promoters, pR1 and pR2. Transcription from pR2 is also induced during the stationary phase and is dependent on RpoS, although probably in an indirect manner [36]. Thus, the following regulation model was proposed: in exponentially growing cells, the balanced growth conditions inhibit the β-ketothiolase activity which is present, and there is also a low transcription of *phbBAC *caused by both the lack of RpoS, which affects transcription from one of the promoters of the PHB biosynthetic operon, and by the low concentraction of PhbR, whose transcription is also partially dependent on this sigma factor [36]. On entering into the stationary phase, the increase in transcription of *rpoS *and consequently of *phbR*, stimulates transcription of the *phbBAC *operon. In addition, the tricarboxylic acid cycle activity may slow down during the stationary phase, allowing for an increase in the acetyl-CoA/CoA ratio, which relieves the inhibitory effect on β-ketothiolase.

The two-component global regulatory system formed by the sensor kinase GacS [32] and its corresponding response regulator GacA [33] is also involved in the control of PHB production in *A. vinelandii *(Figure 2). Either *gacS *or *gacA *mutations diminish PHB production. The model proposes that GacA plays a role as a positive regulator of PHB synthesis in its phosphorylated form. GacA is required for transcription of *rpoS *[33]. Hence, at least part of the control that this system exerts on PHB production can be explained by its effect on the expression of the sigma factor RpoS.

The nitrogen-related phosphotransferase system (PTS^Ntr^; Figure 2), a homolog of the phosphoenol pyruvate-sugar phosphotransferase system (PTS) which mediates the uptake and concomitant phosphorylation of glucose and other carbohydrates in a number of bacterial genera [39] is also involved in the control of PHB accumulation in *A. vinelandii*. A mutation on *ptsP*, encoding enzyme I^Ntr^, lowers the accumulation of PHB [6]. This regulation is probably exerted through a phosphate relay, where enzyme I^Ntr ^autophosphorylates using phosphoenolpyruvate, and IIA^Ntr ^protein appears to be the terminal phosphoryl acceptor (and acts as a negative regulator of PHB synthesis (G. Espín, unpublished data).

It has also been argued that the Fnr-like regulatory protein called CydR may control PHB synthesis in *A. vinelandii *[40]. CydR acts as a repressor in the transcription of *cydAB*, the genes coding for the cytochrome *bd *terminal oxidase required for aerotolerant nitrogen fixation. The DNA binding capacity of CydR is diminished in the presence of oxygen, and transcription of *cydAB *is derepressed [41]. While looking for CydR regulated genes, Wu et al. [40] found that a *cydR *mutant overexpresses β-ketothiolase and acetoacetyl-CoA reductase, and accumulates PHB throughout the exponential growth rate. It is probable that the role of CydR in the control of PHB synthesis is related to the redox state of the cell. However, the mechanism used and its relationship with other regulatory systems is unclear.

## 3. Use of mutants for modifying alginate characteristics or for improving alginate production

Genetic strategies for the construction of *A. vinelandii *mutants have proven to be useful for the elucidation of regulatory aspects in the biosynthesis of alginate and therefore for the construction of *A. vinelandii *mutants exhibiting higher specific alginate production. Moreover, it has been useful for generating strains which produce alginates with specific chemical characteristics. The rationale for the construction of such mutants is discussed in the following section and the main results in terms of the alginate concentration and its molecular characteristics are also summarized in Table 1 for cultures in shake flasks, and in Table 2 for cultures carried out in fermentors.

**Table 1:** Maximal alginate concentration, alginate yield, broth viscosity and molecular weight of alginate produced with different strains of A. vinelandii*.

Strain	Algin_max _(g/L)	Yield (g g-1)	Viscosity_max _(cps)	Molecular weight (kDa)	References
ATCC9046	5.0	1.2	500–700	1500–1900	[45, 78]
CN26	6.0	1.16	400	1130	Unpublished data
DM	4.5	1.3	619	1700	Unpublished data
AT268	6.5	1.8	700	1100	Unpublished data
SML2	6.6	1.9	435	1150	[16]
					
AT6	2.1	3.02(g alg/g prot)	ND	340	[7]

*Cultures conducted in shake flasks at 200 rpm and 29°C.

**Table 2:** Maximal alginate concentration, alginate yield, broth viscosity and molecular weight of alginate produced with different strains of A. vinelandii*.

**Strain and Conditions**	Algin_max _(g/L)	Yield (g g-1)	Viscosity_max _(cps)	Molecular weight (kDa)	References
**3% DOT 300 rpm**					
ATCC9046	4.0	1.14	24	770	[47]
CN26	2.5	0.38	9	400	[4]
DM	2.5	0.83	130	4000	[4]
AT268	3.0	1.0	52	800	[4]
SML2	2.6	0.4	60	1240	[16]
					

					
**3% DOT 700 rpm**					
ATCC9046	3.5	1.2	ND	1250	[69]
					
SML2	3.5	0.9	ND	985	[69]
					
No-control of DOT (340 RPM)					
ATCC9046	8.0	1.5	550	1100	[78]

*Cultures conducted in fermentor at 300 and 700 rpm constant.

### 3.1 Alginate production by *A. vinelandii *mutants with increased algD transcription

As previously discussed, an extensive work has been carried out, in order to unveil the regulatory network which controls alginate synthesis in *A. vinelandii *[24,28-33,42,43]. The global regulators GacA/GacS and the sigma factor AlgU seem to represent key elements in this regulation, and the *algD *promoters are targets in *A. vinelandii*. A fact which upholds this observation is that transcription of the *A. vinelandii algD *gene correlates with the production of alginate [22,31,42]

*mucA *and *mucABCD *mutations increased *algD *transcription two and four-fold, respectively [31]. When transferred to the wild type AEIV, a non-highly mucoid strain, these mutations increased alginate levels up to six-fold. However, in the background of the highly mucoid strain ATCC 9046, only a minor increment of 39 % (for the *mucA *mutation) and 85 % (for the *mucABCD *mutation) was observed. To our knowledge, the *A. vinelandii *ATCC 9046 *mucABCD *mutant derivative named JRA4, showed the highest level of specific alginate production (8.9 mg of alginate/mg of protein), ever reported for this bacterium. However, spontaneous derivatives with reduced levels of alginate production (2.5 mg/mg of protein) appeared at a high frequency, implying that elevated AlgU activity and/or increased alginate production are deleterious to *A. vinelandii *[31].

Random Tn5 mutagenesis of WI12 strain, an ATCC 9046 derivative carrying an *algD::lacZ *transcriptional fusion was carried out [42]. Two mutants called AC28 [42] and RC26 [4], which showed a 3 and 1.9-fold increase in levels of *algD *transcription respectively were identified. The Tn5 mutations in strains AC28 and RC26 were shown to reside within the *ampDE *operon (*muc28 *mutation) which participates in the intracellular recycling of the cell wall, and within a gene named muc26, which encodes a conserved hypothetical protein (Gene Object ID 400259050) of the *A. vinelandii *genome project [44], respectively. The double *ampDE *mutation, but not the single *ampD *or *ampE *mutations exhibited a three-fold increase in *algD *transcription. Transfer of the *ampDE*::Tn5 mutation to the ATCC 9046 strain was unsuccessful. However, when transferred to the AEIV strain, this mutation was viable and increased alginate production eight-fold.

The *muc26 *mutation was transferred to wild type strain ATCC 9046 in order to produce mutant CN26. Although a high alginate concentration (7 g/L) was obtained in the culture of this mutant in shake flasks (Table 1), under controlled conditions of pH (7.2), agitation rate (300 rpm) and dissolved oxygen tension (DOT) (3% DOT), this muc26 mutant did not improve the volumetric production of alginate when compared to the wild type strain ATCC 9046 (Table 2) [4].

Since alginate and PHB are two polymers which compete for the carbon source, we hypothesized that *phbR *mutation would improve alginate yield in the *muc26 *mutant background. Therefore, a double mutant (*muc26 phbR*) was constructed, and named DM [4]. No increase in alginate production was apparent; however, a significant increment in molecular weight (from 0.8 × 10^6 ^to 4.0 × 10^6 ^Da) of alginate produced by strain DM was observed, when the cells were cultured under controlled DOT conditions (Table 2). This is the highest which has been reported for a bacterial alginate. This value is higher than that of a commercial alginate from *Macrosystis pyrifera *(1.1 × 10^6 ^Da) and that of the alginate produced by the *A. vinelandii *parental strain in shake flasks (1.9 × 10^6 ^Da) [45]. The polymer produced by any of the single mutants, *muc26 *(CN26 strain) or *phbR *(AT268 strain) showed wild type characteristics (Table 2). Since the mechanism determining the molecular mass of alginate is poorly understood and since the *muc26 *mutation interrupted a gene encoding a hypothetical protein, it is difficult to offer an explanation for the high molecular weight exhibited by the alginate produced by the DM mutant.

### 3.2 A phbBAC mutant increases alginate production

*A. vinelandii *converts carbon substrates to alginate and PHB, thus the synthesis of PHB is undesirable when optimizing alginate production. Strain AT6, a derivative of ATCC 9046, carrying a *phbBAC *mutation which abrogates PHB synthesis, was shown to increase the alginate yield up to a value of 3.02 g alg/g protein (Table 1), presumably as a consequence of the greater availability of carbon source. However, the volumetric yield of alginate was not significantly increased due to a deleterious effect of this mutation upon cell growth [46].

Taken together, these results suggest that the use of *A. vinelandii *ATCC 9046 mutant derivatives with higher levels of alginate, due either to an increase in AlgU activity or to an increase in the available carbon source seems not to be feasible for industrial purposes and suggests that a different approach must be taken in order to improve volumetric alginate yield in this *A. vinelandii *strain. This fact is further supported by a previous suggestion that strain ATCC 9046 contains a *muc*-1 mutation which increases AlgU activity and consequently alginate production [29]. Therefore, it is likely that the metabolic limits for alginate production have already been reached for the ATCC 9046 strain and that any other mutation increasing alginate production will prove either to be unviable, or to be viable, but at the expense of cell growth.

### 3.3 Alginate lyase and its role in the molecular weight of alginate

The rheological and gel-forming properties of alginate are largely dependent on the molecular mass distribution (MMD) and the relative content of D-mannuronic and L-guluronic monomers [1]. Oxygen concentration has been shown to influence the molecular mass of alginate produced by *A. vinelandii *[47]. A high molecular mass alginate can be produced at a DOT of 3 %. However, a drop in the mean molecular mass of alginate was observed towards the end of the incubation period, presumably as a consequence of a de-polymerization activity carried out by an alginate lyase [47]. To investigate the effect of *A. vinelandii *AlgL on the molecular weight of alginate, a non-polar *algL*::Gm mutant was constructed, and was named SML2. No alginate lyase activity was detected in the SML2 strain. Since AlgE7 epimerase exhibits alginate lyase activiy *in vitro *[48], it is possible that this activity is either very low or is only expressed under specific conditions. The specific production of alginate by the SML2 strain was reduced 35 %, when the cells were cultured in a fermenter at 3 % of DOT and 300 rpm, perhaps as a consequence of the gentamycin cassette insertion which might have had some polar effect on genes downstream *algL*. In contrast to the wild type strain, in *A. vinelandii *SML2 cultures, no drop in the MMM was observed (Table 2), indicating that AlgL was responsible for the de-polymerization of alginate [16].

### 3.4 Alginate acetylation

Bacterial alginates differ from algal alginates because of the presence of O-acetylated mannuronate residues; the majority of these residues are mono-O-acetylated but a few are 2,3-di-O-acetylated [49]. In an attempt to obtain an *A. vinelandii *mutant producing non-acetylated alginate strain, an *algF*::Tc non-polar mutant was constructed and was named AJ34. This mutant produced a non-acetylated alginate which appeared to confer a rough phenotype to the colony and a reduction in the formation of cysts resistant to desiccation [27]. Alginate production by AJ34 strain mutant was reduced by 50 %, probably due to a polar effect on *algA *transcription from the tetracyclin cassette promoter [27]. Further work is needed in order to assess the rheological characteristics of this polymer and to investigate whether these non-acetylated alginates constitute better substrates for the *A. vinelandii *AlgE1-7 epimerases, which only modify non-acetylated M residues.

### 3.5 *A. vinelandii *epimerases

As pointed out previously, alginates are composed of variable amounts of (1–4)-β-D-mannuronic acid (M residues) and its epimer, α-L-guluronic acid (G residues). Their relative content and sequence distribution vary widely and have profound effects on the physicochemical properties of the polymer. In *A. vinelandii*, a family of seven secreted and Ca++ dependent C-5 epimerases (designated AlgE1-AlgE7) have been identified and these are responsible for generating a variety of epimerization patterns, including G blocks of various lengths [50]. Each enzyme is composed of two structurally distinct modules, designated A and R. The A modules (about 385 amino acids) are present in one or two copies in each enzyme, while the R modules (about 155 amino acids) are present in between one and seven copies. It was shown that the A module alone is sufficient for both the epimerization, as well as the determination of sequence distribution [51]. G blocks are of great biological and biotechnological significance as they are a prerequisite for the formation of strong polymer gels in the presence of divalent cations like Ca2+; however the composition of the commercially available alginates vary, depending on the source of the polymer. Hence extensive work has been carried out in order to investigate the epimerization properties for each *A. vinelandii *C-5 epimerase, with the aim of increasing the G content of commercially available alginates, containing a wide range of initial G residues, or in order to produce alginates with specialized properties [50,52,53]. While algE4 activity introduces alternating M and G residues into substrate, the remaining six enzymes introduce a mixture of continuous stretches of G residues and alternating sequences. Furthermore, Bjerkan et al. [52] constructed hybrid mannuronan C-5 epimerases, by exchanging parts of the sequences encoding the A module of AlgE2 (which generates consecutive stretches of G residues) and AlgE4 (which generates alternating structures). These hybrid enzymes introduce a variety of new monomer-sequence patterns into their substrate. Those authors also identified some regions of the A module, important for the specificity or processivity of the enzymes. These studies, besides helping to elucidate the structure-function relationship for each enzyme, open up new possibilities for biotechnological applications.

## 4. Mutations which increase PHB production

### 4.1 Blocking alginate synthesis, promote PHB accumulation

Alginate synthesis may constitute a waste of substrate when seeking to optimize PHB production. The effects on PHB accumulation of two mutations causing different blockades in the alginate biosynthetic pathway have been described. Martínez et al. [54] evaluated the effect of a mutation in the *algK *gene on PHB production (Figure 1). This gene encodes a protein which is probably involved in guiding alginate for secretion, and protecting it from AlgL degradation [17,55] and whose inactivation impairs alginate production [26]. When compared to that of the wild strain, PHB accumulation of the algK mutant, increased by 50 %, measured as milligrams per milligram of protein. The *algK *mutant also showed a 50 % higher yield of PHB per sucrose consumed. However, the possibility that this strain might still have the potential to drain carbon for the synthesis of alginate precursors was raised. Evidence supporting this theory is derived from the description of a *P. aeruginosa algK *mutant| which is also unable to produce alginate and has been shown to secrete uronic acids [56]. These acids seem to be the products of alginate degradation by AlgL [17].

**Figure 3:**
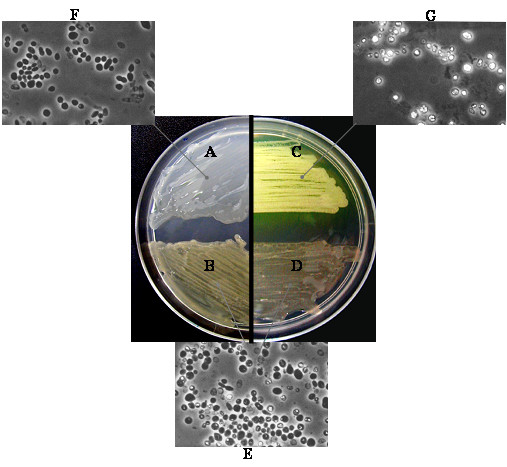
Alginate production (mucoidy; A, B) and PHB accumulation (opacity; C. D) of *A. vinelandii *wild type ATCC 9046 and mutant strains grown on plates containing sucrose as carbon source. The higher mucoid phenotype of strain AT6 (A) compared to ATCC9046 (B), shows the alginate overproducing phenotype of this strain unable to produce PHB. The absence of PHB granules in AT6 is shown in (F), as compared to ATCC9046 wild type (E). The increased opacity phenotype of the non-mucoid strain AT41 (C) when compared to ATCC 9046 (D), is due to the overproduction of PHB. The high content of PHB granules can be observed in the bright AT41 cells (G).

A more recent work [46] studied the effect of a blockade on the first enzymatic step of the alginate biosynthetic pathway, the phosphomannose isomerase (Figure 1), on PHB production. This enzyme, together with the guanosine diphospho-D-mannose pyrophosphorylase (third step of this pathway) is encoded by the *algA *gene as a bifunctional enzyme. The *algA *mutation (strain AT41, Figure 3) impaired alginate production and increased PHB accumulation (in grams of PHB per gram of protein) between 75 % and 500 %, depending on the medium used, with a 61 % higher yield (gram of PHB per gram of glucose consumed). It is interesting to note that the *algA *mutation not only increased the capacity of the bacterium to produce more PHB per biomass unit, but also permitted better growth of the mutant, influencing the volumetric production of PHB and improving it up to 10-fold.

### 4.2 The lack of the anaplerotic enzyme pyruvate carboxylase promotes PHB accumulation

An article published recently [57] describes another interesting mutation found to positively affect PHB accumulation capacity. A strain accumulating 3.5 times more PHB (grams of PHB/gram of protein) than its parental strain, a non-alginate producing strain was identified, by mini *Tn5 *mutagenesis. The Tn5 insertion knocked out a gene (*pycA*) coding for a subunit of the pyruvate carboxylase. This enzyme catalyzes the ATP-dependent carboxylation of pyruvate, to generate oxaloacetate which replenishes the tricarboxylic acid cycle, during cell-material biosynthesis (Figure 1). The authors hypothesized that the knocking out of *pycA *results in a lower oxaloacetate pool, and as the flux of acetyl-CoA in the TCA cycle depends on this being condensated with oxaloacetate to produce citrate, the result is that the acetyl-CoA remains available for PHB synthesis, inducing a high PHB accumulation. A low carbon flux throughout the TCA cycle would not only favor PHB accumulation due to a higher acetyl-CoA availability, but would also diminish the CoASH pool, favoring β-ketothiolase activity.

### 4.3 A defect in the respiratory NADH oxidase improves PHB synthesis

Several years ago another mutation affecting central metabolism was reported as positively affecting the PHB accumulation capacity in *A. vinelandii*. Page and Knosp [58] reported the isolation of *A. vinelandii *UWD, a mutant which produces PHB during exponential growth, without requiring nutrient limitation. The reason for this phenotype was apparently a defect in the respiratory NADH oxidase, which increases NADH concentration, lowering the citrate synthase activity. The PHB production increased 2.6 to 3.4 fold, measured as a percentage of the dry weight basis, and the PHB yield (grams of PHB/gram of glucose consumed) increased 5 – 6.6 fold.

## 5. From plate to fermentor

One of the aspects frequently considered during the selection of polysaccharide-overproducing strains on plates is the mucoid phenotype of the colonies. This is the case for the *A. vinelandii *mutant strain impaired in PHB synthesis (AT6). This strain was isolated in our research group and presented a highly mucoid phenotype when compared to the parental strain (ATCC 9046) (Figure 3). It is important to point out that mucoidy is determined both by the concentration of the polymer and/or its chemical characteristics, specifically its molecular mass. For this reason, both characteristics were evaluated in plates as well as in submerged cultures. Although the mutant AT6 showed a high yield in plate as well as in submerged culture [46], the alginate volumetric production and the molecular mass of the polymer were lower than the values exhibited by the parental strain (Table 1).

A similar problem can be observed when seeking for improved strains for the purpose of PHA production. The *A. vinelandii *pyruvate carboxylase mutant (AJ1678), characterized as PHB overproducer strain, was identified because its colonies have a higher opacity on plates containing sucrose. As expected, the opacity was due to a higher amount of PHB granules in the cells. However, when this strain was grown in shake flasks, it produced the same amount of PHB as that obtained using the wild type, probably due to the presence of the alternative enzyme, PEP carboxylase, under this condition [57].

Overall, our results reveal the limitations of selecting alginate or PHB over-producing strains, using only the phenotype observed on plates as criterion and emphasize the necessity to characterize the mutant strains, using controlled culture conditions.

## 6. Improving quality and quantity of alginate by means of fermentation strategies

Several studies were carried out at the beginning of 80's, which described alginate production by *A. vinelandii *either in batch [59,60] or continuous cultures [61-63]; however to our knowledge, none of these processes has yet been adopted for the industrial production of microbial alginates. Using bioengineering tools, several new experimental strategies were reported, only recently. These make it possible to obtain higher yields of alginates, with certain characteristics which are suitable for particular applications and are thus more competitive for the microbial polymer market. In this section we will describe and discuss the most recent advances regarding the influence of fermentation parameters, which determine the production and composition of alginate, as well as the few reports about the scale-up of the process and novel fermentation strategies for the production of alginate.

### 6.1 Influence of dissolved oxygen tension (DOT) and mixing

Aeration and mixing are critical parameters for the optimal production of polysaccharides. Reports have been published about the influence of these parameters on the concentration and chemical characteristics of the alginate, synthesized by *A. vinelandii *[47,64,65]. It is important to point out that dissolved oxygen tension (DOT) can be controlled, either by manipulating the agitation rate of the culture, or by varying the proportions of nitrogen or oxygen present in the gas inflowing through mass flow controllers [47,66]. The advantage of this latter method is that it is possible to independently evaluate the effects of hydrodynamics and oxygen transfer conditions. In the previous reports (cited in previous reviews [67,68]) the dissolved oxygen tension (DOT) was controlled by varying the agitation rate of the bioreactor and, for this reason, it was not possible to discriminate between the influence of the oxygen in the bulk liquid and the agitation speed, on alginate production.

Data obtained under non-nitrogen fixing [47,66,69] and nitrogen-fixing conditions [64], indicate that alginate production, as well as the molecular mass of the polymer, are strongly influenced by dissolved oxygen tension (DOT) and the stirring speed of the culture. Peña et al. [47] have found that under high DOT (5 % of air saturation), the bacteria produced more alginate (4.5 g/L) than that obtained at low (0.5 %) oxygen tension (1.0 g/L) in cultures conducted at 300 rpm. On the other hand, the higher the stirring speed (from 300 to 700 rpm), the higher the specific growth rate and alginate production rate in cultures where the DOT was constant at 3 %. However, low agitation speed (300 rpm) lead the culture to produce a polymer of high molecular mass (680 kDa), whereas a low molecular mass (352 kDa) alginate was obtained from cultures conducted at high (700 rpm) stirring speed. At 700 rpm, the mean molecular mass (MMM) increased to a plateau between 1 and 3 % DOT and then decreased to a minimum of 0.11 × 10^6 ^g/g mol at 7 %. Microscopic observations revealed the presence of cellular aggregates when the culture was conducted at 300 rpm. Oxygen gradients occurring within big aggregates (more than 1000 μm) may be responsible for this phenomenon [70]. It is important to point out that with a high agitation rate, the MMM of the alginate dropped towards the end of the culture in all conditions evaluated, which was probably due to AlgL activity [47].

Sabra et al. [64] reported that in phosphate-limited continuous culture, the specific rates of oxygen consumption and alginate formation of *A. vinelandii *increased as a function of the DOT of the culture, obtaining a specific alginate production rate of 0.2 g/g.h at a dilution rate of 0.22 h^-1 ^at 5 % of DOT [64]. Furthermore, in the same study, the authors reported that both the molecular mass and the L-guluronic acid content increased with the DOT, reaching a maximal molecular mass of 800 kDa and a guluronic acid content of 50 % in the cultures conducted at 10 % of air saturation. Sabra et al. [64] proposed that under nitrogen-fixing conditions, the bacterium builds an alginate capsule, with the composition varying in accordance with the external DOT and this may also help to protect the nitrogenase system against oxygen damage.

**Figure 4:**
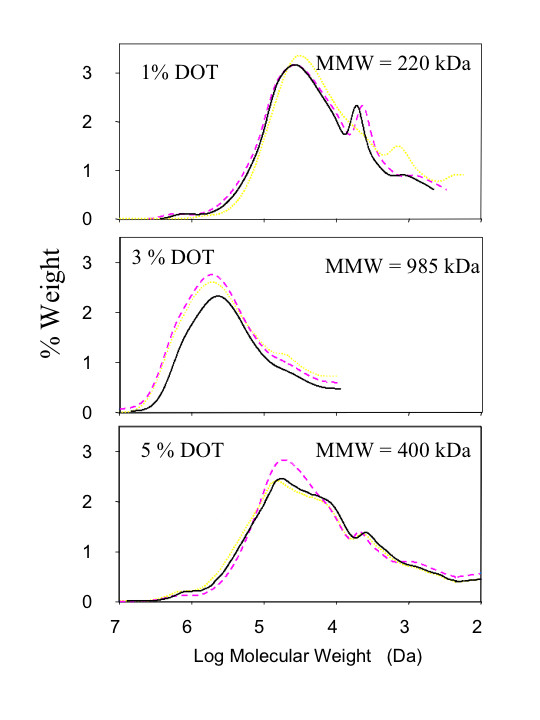
Influence of the dissolved oxygen tension (DOT) on the molecular weight distribution of the alginate obtained with *A. vinelandii *SML2 strain. Dotted line 7 h, dashed line 13 h, solid line 21 h y 16 h (for 1% and 3–5 % of DOT respectively). Taken with permission from Trujillo-Roldán et al. [69].

Trujillo-Roldán et al. [69] have made clear that alginate polymerization occurs by making chains with very uniform molecular mass distributions, which have low dispersion throughout the culture, regardless of the strain used (wild type or AlgL mutant) and of culture time (Figure 4). In addition, the MMM of these families is strongly affected by DOT, increasing to a plateau (between 1 and 3 % of DOT) and then decreasing at higher DOT values (Figure 4). These results indicate that the polymerase is highly affected by DOT. It is possible that transcription of *alg8*, encoding the polymerase, is affected by DOT as is the case for *algA, algC and algD *transcription in *P. aeruginosa *[71]. Trujillo-Roldán et al. [69] reported that the alginate-lyase is not essential for the production of alginate; however, when the enzyme is present (as in the wild type), its role is restricted to a post-polymerization step, with its activity reaching a maximum in the pre-stationary phase of growth. The action of alginate-lyase is evidenced by a drop in the MMM of the alginate families.

### 6.2 Influence of medium components

It has been reported [67] that the components of the culture medium play an important role in determining the alginate production in the case of *A. vinelandii*. Recent literature has focused on the study of phosphate and nitrogen and how these affect the concentration and quality of the alginate produced [65]. According to these authors, alginate production was not affected by phosphate and nitrogen concentration. In addition, they reported that the depolymerization of the alginate may be related to the concomitant occurrence of two or more limitations (low levels of oxygen, nitrogen or phosphate) or to the energetic state of the cells.

Peña et al. [3] have reported how (3N-morpholino)-propane-sulfonic acid (MOPS), a component used in the medium in order to keep the pH constant during cultivation of *A. vinelandii *influences the quality of the alginate in terms of its chemical composition and also the rheological behavior of alginate-reconstituted solutions. This compound had an important affect on the acetyl content of the alginate and in turn on the physico-chemical properties of this polymer. A higher acetylation of alginate was obtained when 13.6 mM of MOPS was supplemented to the medium. This value was twice as high as that obtained when no MOPS was used. The higher acetylation resulted in greater viscosity in the alginate solutions, but it exhibited less pronounced pseudoplastic behavior. These changes in the functional properties of the polymer can have great value in terms of specific applications of alginate in food and pharmaceutical fields.

The inoculation process represents an important aspect during alginate production, even though it is generally considered to be irrelevant. A typical inoculation consists of a pre-culture consisting of between 1 and 20 % (v/v) of the working volume of the production fermenter, where the exhausted culture medium is added to the new medium in the fermenter, together with bacterial cells. By washing the cells prior to inoculation, Trujillo-Roldán, et al. [72] have shown that it is possible to obtain alginates of a higher molecular mass (1200 kDa) than those obtained in cultures conventionally inoculated (350 kDa). These results suggest that components in the exhausted inoculum broth affect alginate characteristics, and should therefore be considered in strategies designed for alginate production.

### 6.3 Use of CO_2 _to prevent alginate degradation

*A. vinelandii *is a bacterium exhibiting a high respiration rate and thus also a high CO_2 _generation rate. A study about the influence of carbon dioxide on the production and quality of alginate in batch cultures, conducted in a 1 L bioreactor under constant dissolved oxygen tension of 3 %, was carried out by Seáñez et al. [73]. Bacterial growth and alginate production were affected by the CO_2 _addition. In terms of growth rate and alginate production, inhibitory (0–8 %) and stimulatory effects (13 %) were observed, and a total growth inhibition was obtained when using 25 % CO_2 _in the inlet gas stream. Studies about the de-polymerization of alginate using broth extract from cultures developed with and without CO_2 _showed that high CO_2 _concentrations inhibit either the synthesis or the activity of AlgL [73]

### 6.4 Novel fermentation strategies

Saude & Junter [74] have reported the production of alginate by batch cultures of *A. vinelandii*, immobilized in a system constituted by a gel layer and a microporous membrane structure. The immobilization of *A. vinelandii *cells favored the production of alginate with a high molecular mass (MM) and low polydispersity, as compared to conventional free-cell cultures grown in shake flasks. Cheze-Lange et al. [75] reported the advantages of continuous production of bacterial alginate by *A. vinelandii*, coupled to a system of membranes of varying nominal pore sizes. According to these authors, the yields of alginate with respect to sucrose were significantly higher than in the batch process; however, the molecular mass of the polymer and the polydispersity were very similar to those of the alginate obtained from the batch experiments.

Asami et al. [76] studied the behavior of alginate synthesis by *A. vinelandii *in batch experiments conducted in bubble column and shake flasks. They found that the productivity and the fraction of GG-blocks of the alginate obtained in the bubble column were higher than those obtained in the shake flasks. In the bubble column, the production of GG-blocks in the late exponential growth phase was higher than that obtained in the stationary phase. However, the authors did not explain the reasons why the fraction of GG blocks changed under varying conditions, for example because of shear stresses and oxygen tension.

Priego et al. [77] used exponentially fed-batch cultures with the aim of determining the effect of specific growth rate on alginate production and on its molecular characteristics. In this study, particular care was taken in terms of the experimental conditions in order to study only the effect of μ, whilst discriminating the effect of other culture variables. The conclusion reached from this study was that the specific growth rate of *A. vinelandii *negatively affects the molecular mass of the alginate and to some extent, the alginate/biomass and alginate/sucrose ratio. This effect was particularly pronounced at very low specific growth rates (0.03 h^-1^) where the Yp/x, Yp/s and MMM increased by up to 2.3, 10 and 14 times higher, respectively, than those obtained at a specific growth rate of 0.21 h^-1 ^(such as that found in conventional batch cultures). These findings are highly relevant for the reliable production of high molecular mass alginates.

### 6.5 Scaling-up of alginate production

The transfer of results obtained in plate to shake-flasks and in turn to stirred tank fermentors is troublesome and in general, poorly understood. There are very few reports covering aspects referring to the scale-up of the process for alginate production. Trujillo-Roldán et al. [66] reported scale-down studies, where conditions occurring in large scale fermentors were simulated in laboratory fermentors. In this study, *A. vinelandii *was cultured under DOT oscillating conditions, in fermentors. Exposure to oscillating DOT with wave periods of 1200 and 2400 s only slightly affected the growth of *A. vinelandii *and alginate production. In contrast, small changes to the average amplitude of the wave drastically affected alginate mean molecular mass and its distribution. These data suggest that poor DOT control in alginate fermentation, caused for example by high viscosity and/or insufficient mixing, could lead to the loss of polymer quality in terms of its molecular weight.

**Figure 5:**
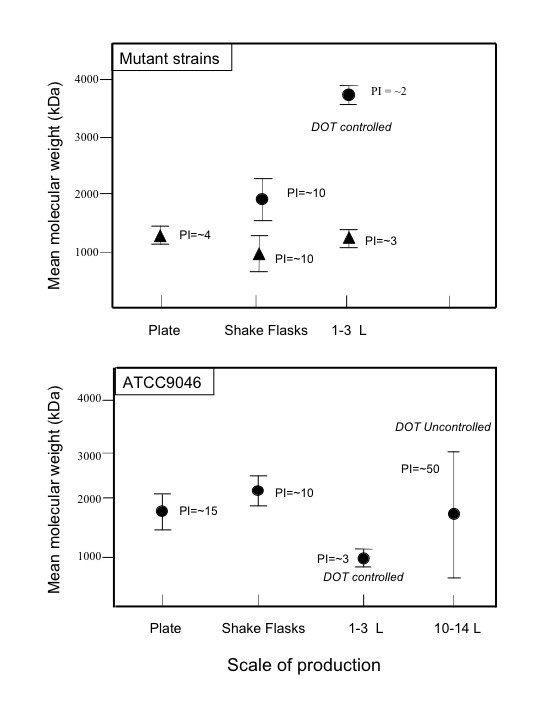
Effect of the scale of production on the mean molecular mass and polidispersity of the alginate obtained with the parental strain (ATCC9046) and various mutant strains. Circles: DM; Triangles: SML2.

The mean molecular mass of alginates produced by *A. vinelandii *in shake flasks can reach values of up to 1900 kDa and viscosities of up to 520 cps, for broths containing about 5 g L^-1 ^of alginate [78]. However, when the process has been translated to laboratory fermentors (1 L), in which pH and DOT were kept constant, the molecular mass and viscosity of the broths were considerably lower, obtaining alginates with a molecular mass of less than 0.68 × 10^6 ^Da and viscosities lower than 100 cps for an alginate concentration of around 5.0 g L^-1 ^[47,66,73] (Figure 5).

Using the specific power consumption (P/V) as criterion, the study carried out by Reyes et al. [78] revealed that in order to scale-up from flasks to fermentor, the initial power drawn did not permit the behavior of shake flask cultures to be reproduced (particularly broth viscosity-concentration profiles and mean molecular mass). Drastic differences in the power drawn evolution may be occurring during the cultures developed in shake flasks and the stirred-bubbled fermentor. Decreasing initial P/V in the fermentor or during cultivation, permitted the molecular characteristics of the alginate obtained in shake-flasks to be matched [78] (Figure 5).

More recently, Peña et al. [79] have rigorously studied both the evolution of the specific power consumption and the oxygen transfer rate, occurring in shake flasks in cultures of *A. vinelandii*, with the purpose of better understanding the behavior of alginate production in shake flasks and in order to develop strategies for the scaling-up of the process. These studies revealed that power consumption increased exponentially during the course of fermentation (up to 1.4 kW m^-3^) due to an increase in the viscosity of the culture broth. At the end of fermentation, when the viscosity and alginate concentration reached a maximum, a slight drop in the power consumption was observed. It is important to point out that the analysis of molecular mass distributions of the alginates suggests that in the shake flask cultures, DOT conditions may be more homogeneous than those present in a stirred fermentor, where control of DOT and pH is lacking [79].

## 7. Fermentation strategies for the PHB production using *A. vinelandii*

*A. vinelandii *usually produces PHB. This polymer forms highly crystalline solids, resulting in the production of brittle plastics [8]. However, by using a fermentation strategy consisting in the addition of valerate, heptanoate or nonanoate to a culture of *A. vinelandii *UWD grown in glucose, Page et al. [80] were able to obtain the copolymer poly(Hydroxybutyrate-Co-Hydroxyvalerate) (P(HB-co-HV)), which produces plastics exhibiting better mechanical properties. These authors reported β-hydroxyvalerate contents ranging from 8.5 to 52 mol %, depending on the concentration of valerate used.

Another aspect which has been evaluated, regarding the quality of PHAs produced by *A. vinelandii *refers to the degree of polymerization. This characteristic is expected to affect the mechanical properties of the plastics produced [81]. In the *A. vinelandii *strainUWD the formation of a very high molecular weight PHB (4 million Daltons) is promoted by some of the non-sugar components of beet molasses. In fact, the molecular weight of PHB can be altered between one and four million Daltons depending on the nature of the carbon source used [81].

A wider use of PHA-derived plastics has been hampered because of their high production costs [82]. The cost of the carbon source contributes significantly to the overall production cost of PHAs [82]. Because of their low price, unpurified organic wastes from agriculture and food processing can be excellent substrates for the bacteria. On this regard, Page [58], reported that the *A. vinelandii *strain UWD is able to grow and produce PHB up to 2.5 g/L using glucose and it can also produce the polymer from fructose, sucrose, maltose, gluconate or glycerol as carbon sources. However, unrefined carbon sources such as corn syrup, cane molasses, beet molasses, or malt extract, also support PHB formation, obtaining yields of PHB comparable to, even better than the refined sugars. Beet molasses and malt extract promoted higher polymer production per liter (2.74 and 2.80 g/L respectively) due to a growth stimulatory effect [58]. The addition of valerate in a fed-batch fermentation using beet molasses as carbon source, sustained the production of (P(HB-co-HV) with a 20 % of hydroxyvalerate, demonstrating that the production of copolymers is also feasible using unrefined substrates [83]. It has been reported that PHA production can be increased and the yield improved, by supplementing a small amount of complex nitrogen sources [80]. The supplementation with fish peptone, proteose peptone, and yeast extract promoted a significant increase in the production of PHB per liter (up to 7.5 g/L). An alternative approach which consisted of supplementing fish peptone to a fed-batch culture, resulted in a high PHB concentration of 32 g/L after 47 h of culture [84].

Another factor influencing production costs is the recovery of the product. Page and Cornish [84] reported that *A. vinelandii *strain UWD cells, when cultured in medium supplemented with fish peptone, become fragile and break easily. Therefore, a simple treatment with 1 M NH_4_OH allows the separation of a highly pure PHA. This phenotype has permitted the development of an economical recovery method [84]. Using a two stage fermentation process as a strategy for improving the production of PHAs. Chen and Page [85] increased the concentration of the polymer produced by up to 36 g/L (in a 2.5 liter fermentor) and also notably improved productivity, by up to 1.05 g/L * h. This process was designed using aeration to promote growth and to suppress PHB production in the first phase, while lower aeration of a culture containing fish peptone as a nitrogen source was used to promote PHB formation in the second phase, taking advantage of the higher biomass achieved.

## 8. Conclusions and future prospects

The production of alginate in *A. vinelandii *seems to be linked to metabolic signals indicating cell damage. This observation is supported by the fact that stress sigma factors AlgU and RpoS are key regulators for alginate synthesis and that a signal derived from cell wall damage triggers alginate production [30,33,42]. Other mutations which have a stimulatory effect on alginate yield (per cell basis), such as the one blocking PHB synthesis [46], also have a negative effect on the growth capacity of *A. vinelandii*. Therefore, it is likely that any mutation increasing alginate production will have a negative effect upon cell growth, and consequently on the volumetric yield of alginate. It will thus be necessary to implement a different approach, in order to overcome this fact. One of these strategies might consist in developing new fermentation schemes, such as multistage fermentations, which promote better growth, in order to take advantage of the higher specific alginate production capacities of such strains. Other strategies might involve metabolic engineering of *A. vinelandii *in order to improve, for example, the availability of fructose 6-P, the precursor of the activated monomer GDP-mannose, by producing this metabolite directly from glucose-6-P instead of making it from trioses when growing on substrates yielding glucose [86]. On the other hand, it was previously reported that in *P. aeruginosa*, the introduction of multiple copy numbers of *alg8 *dramatically increased alginate production, suggesting that the polymerization step constitutes a bottleneck in the production of alginate [9]. It would thus be interesting to test, whether this is also the case for *A. vinelandii*. The existence of seven C-5 epimerases in *A. vinelandii*, showing a variety of epimerization patterns indicates that this bacterium is capable of producing alginates with a great variety of characteristics [11]. It would be interesting to investigate the environmental conditions which might be affecting these epimerization activities, so as to be able to produce alginates with a specific degree of epimerization and sequence distribution. From the fermentation/bioengineering side, it is evident that relatively minor improvements have been achieved in terms of the volumetric yield of alginate; however significant improvements have been achieved in terms of the molecular characteristics of the polymer, by manipulating environmental conditions. In particular, it has been shown that dissolved oxygen tension and the specific bacterial growth rate play a key role in defining the molecular weight distribution. In addition, the manipulation of culture broth components (such as MOPS) influences the acetylation degree of the polymer. This knowledge opens up many possibilities for designing processes to produce tailor-made alginates. Although recent research concerning fermentation strategies using *A. vinelandii *strains for the production of PHAs production is scarce, a mutant strain of this organism has shown to be potentially useful for the production of PHB and its copolymers [58]. Our understanding about the regulation of PHAs synthesis in *A. vinelandii *and of its metabolic relationships with other pathways has grown considerably. However this information has not been used for designing improved strains or new fermentation procedures in order to increase PHA productivity. It would be interesting to test thoroughly for the PHB production capacity among strains such as the *algA *or the *pycA *mutants, which have been shown to significantly increase the amount of accumulated PHB.
